# ARC/Arg3.1 expression in the lateral geniculate body of monocular form deprivation amblyopic kittens

**DOI:** 10.1186/s12886-022-02757-5

**Published:** 2023-01-03

**Authors:** Haobo Fan, Ying Wang, Yunchun Zou, Weiqi Song, Juan Xie, Xiuping Tang, Siyu Chen

**Affiliations:** 1 Department of Optometry, North Sichuan Medical College, No.234 FuJiang Road, Nanchong, 637000 China; 2 Department of Optometry and Pediatric Ophthalmology, Ineye Hospital of Chengdu University of TCM, Chengdu, China; 3 Department of Ophthalmology, the Second Clinical College of North Sichuan Medical College (Nanchong Central Hospital), Nanchong, China

**Keywords:** Amblyopia, Activity-regulated cytoskeleton-associated (ARC/Arg3.1), Lateral geniculate body, Form deprivation

## Abstract

**Purpose:**

The present study compared the expression of activity-regulated cytoskeleton-associated protein (ARC/Arg3.1) in the lateral geniculate body between form deprivation amblyopia kittens and normal kittens to examine the significance of ARC/Arg3.1 in the lateral geniculate body in the pathogenesis of amblyopia.

**Methods:**

Twenty kittens were randomly divided into an experimental group (*n* = 10) and a control group (*n* = 10). Black opaque covering cloth was used to cover the right eye of kittens in the experimental group. Pattern visual evoked potentials (PVEP) were detected weekly in all kittens. The expression of the ARC/Arg3.1 gene was detected by immunohistochemistry and in situ hybridization, and apoptosis of lateral geniculate body cells was detected by TUNEL.

**Results:**

PVEP detection showed that at the age of 5 and 7 weeks, the latency of P100 in the right eye of the experimental group was higher than that of the other three groups (*P* < 0.05), and the amplitude of P100 was lower than that of the other three groups (*P* < 0.05). The expression of ARC/Arg3.1 protein (*P* < 0.05) and mRNA (*P* < 0.05) in the lateral geniculate body of the experimental group was significantly lower than that of the control group. The level of neuronal apoptosis in the experimental group was higher than that in the control group (*P* < 0.05). The expression of the ARC/Arg3.1 gene was negatively correlated with the apoptosis level of lateral geniculate body neurons.

**Conclusions:**

The expression of ARC/Arg3.1 is associated with monocular form deprivation amblyopia and apoptosis of lateral geniculate body cells.

## Background

Related studies believe that visual developmental plasticity is the basis of amblyopia treatment, and its performance is closely related to synaptic plasticity [1, 2]. During the occurrence and development of amblyopia, the synaptic density in ganglion cells, lateral geniculate body, and visual cortex of animals changed [3–5]. Synapse, as the structural basis of information transmission between neurons, is the key part of visual development plasticity [6]. In many pathogeneses of amblyopia, synaptic plasticity is considered the most critical link and maybe the final pathway of other pathogenesis. Its plasticity can be divided into long-term potentiation (LTP) and long-term depression (LTD) according to time [7, 8].

Immediate early genes also have the effect of coupling short-term signals with long-term changes [9]. As one of the immediate early genes, activity-regulated cytoskeleton-associated protein (ARC/Arg3.1) is induced in neurons in response to neural activity and is necessary for activity-induced forms of synaptic plasticity [10]. Moreover, it also is a crucial regulator of memory and cognitive flexibility [11]. However, there has been no study on the correlation between the expression of ARC/Arg3.1 and amblyopia. Therefore, we examined changes in ARC/Arg3.1 in the lateral geniculate body in amblyopia to investigate the significance of this body in the pathogenesis of amblyopia and provide theoretical support for the occurrence and development of amblyopia.

## Methods

### Animals

We used 20 healthy 3-week-old kittens weighing between 250 and 350 g, regardless of coat color and gender. All kittens were examined to rule out congenital and developmental abnormalities such as refractive media and fundus opacity, and the refractive errors were + 2.25 ~  + 3.50D. All kittens were kept in a room with an ambient temperature set to 24 ± 1 °C and relative humidity of 50 ± 10%, which had great ventilation and natural light. Up to the age of 5 weeks, all kittens were fed milk powder and water 5 times a day as they were unable to feed on their own. After 5 weeks of age, the kitten has fed solid food and drank water three times a day. This study has been approved and supervised by the Experimental Animal Ethics Committee of North Sichuan Medical College (NSMC Appl. No. 2021 [66]), and all animals in this study were provided by the Experimental Animal Center of North Sichuan Medical College.

### Animals model establishment

Twenty kittens used the random number table method to divide into the experimental group (*n* = 10) and the control group (*n* = 10). The kittens were anesthetized by intraperitoneal injection of 1% pentobarbital sodium (35 mg/kg). The right eye of the kittens in the experimental group was covered with black opaque covering cloth, while the control group was only anesthetized. Pattern visual evoked potential (PVEP) examination was performed on two groups of kittens regularly every week. The successful establishment of the amblyopia model was based on the standard that the amplitude of the P100 wave in the right eye (the covering eye) of the experimental group was lower than that of the other three groups, and the latency of P100 wave was higher than that of the other three groups [12–14].

### PVEP detection

All kittens were anesthetized intraperitoneally with 1% pentobarbital sodium (35 mg/kg). The refractive errors of all kittens were detected by retinoscopy and corrected by lenses. Then, the animal electrode needles (RL-1223000030-RC-D, Roland Consult Stasche Finger Gmbh) were inserted into the middle of the forehead, occipital and posterior part of the ear tip of kittens. Place the kitten 40 cm away from the vertical line in the center of the display screen, and adjust the head position so that its visual axis is perpendicular to the screen. Set the program of Reti-Port/Scan 21B (Roland Consult Stasche Finger Gmbh), select the chessboard flip mode, the time–frequency 1 Hz, 0.3 cpd mode, a contrast of 97%, superimposed 64 times and sampling time of 300 ms [15]. The test was repeated 3 times for each eye of each kitten and the average value was obtained.

### Dissection of the lateral geniculate body

**Fig. 1:**
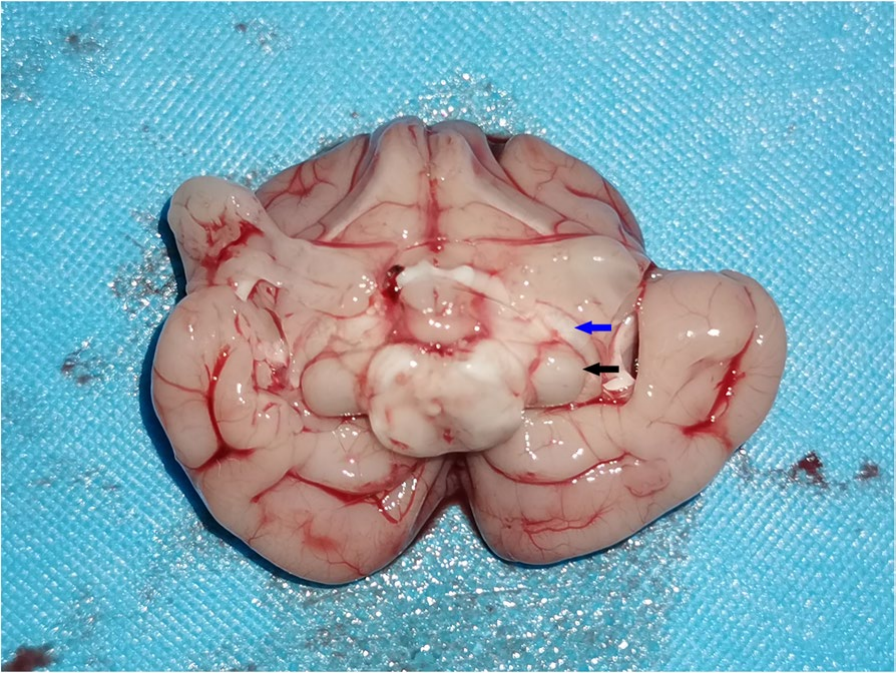
Anatomical schematic diagram of the lateral geniculate body. According to the direction of the optic tract (blue arrow), the lateral geniculate body (black arrow) can be seen and separated

At the age of 7 weeks, after PVEP detection, all kittens were euthanized by intraperitoneal injection of 2% pentobarbital sodium (100 mg/kg) according to the American Veterinary Medical Association (AVMA) Animal Euthanasia Guidelines (2020). According to the Atlas of Feline Anatomy For Veterinarians, the left lateral geniculate body of kittens was isolated and fixed in 4% paraformaldehyde (Fig. 1). Then paraffin embedding was carried out and paraffin sections were prepared. The paraffin section was made by treating the whole tissue block as a whole, and the thickness of the section was set to 4 μm. The expression of ARC/Arg3.1 was detected by immunohistochemistry (IHC) and in situ hybridization (ISH), and the apoptosis of neurons in the lateral geniculate body was detected by TdT-mediated dUTP Nick-End Labeling (TUNEL).

### Immunohistochemical staining

Sections were dewaxed to water and placed in 3% hydrogen peroxide solution and phosphate buffer saline (pH 7.4) (Boster Biological Technology Co., Ltd., China, AR0030) in turn to block endogenous peroxidase. The slices were placed in a repair box containing citric acid (pH 6.0) (Boster Biological Technology Co., Ltd., China, AR0024) antigen repair buffer for antigen repair. The tissue was then evenly covered with 5% BSA blocking solution in the culture dish for serum blocking. Followed by the addition of the first antibody (the dilution ratio of the antibody was 1: 100) (ARC/Arg3.1) (Proteintech Group, Inc, China, 16,290–1-AP). The second antibody (Biotin Conjugated goat anti-rabbit IgG) and strept avidin–biotin complex (SABC) (Boster Biological Technology Co., Ltd., China, SA1022). Diaminobenzidine (DAB) (Boster Biological Technology Co., Ltd., China, AR1022) was used to show color, and positive results ranged from yellow to brownish yellow. The nucleus of hematoxylin staining (Beijing Solarbio Science & Technology Co., Ltd., China, Ltd, G1080) was blue. The tissue was then dehydrated, and microscopic examination, image acquisition, and analysis were performed. Three fields of view were randomly selected for each slice for statistical analysis.

### In situ hybridization staining

Paraffin sections were dewaxed in water and digested with protease K (20 μl/ml) at 37℃ for 30 min. The 3% methanol-hydrogen peroxide was added, and the slide was placed in phosphate buffer saline (pH 7.4) (Boster Biological Technology Co., Ltd., China, AR0033) to block endogenous peroxidase. After pre-hybridization, ARC/Arg3.1 mRNA probe (5`-CGCTG GGTCA AGCGT GAGAT GCACG TGTGG AGGGA-3`; 5`-TATTG GCTGT CCCAG ATCCA GAACC ACATG AATGG-3`; 5`-TGGCG TAAGC GGGAC CTGTA CCAGA CACTG TATGT-3`) hybridization solution containing the probe was added (Boster Biological Technology Co., Ltd., China, MK1612) at a concentration of 20 μl. Hybridization was conducted at 37℃ in an incubator overnight, and then the hybridization solution was washed away. BSA blocking solution was then added, followed by a drop of mouse anti-digoxigenin-labeled peroxidase (Boster Biological Technology Co., Ltd., China, MK1748). DAB (Boster Biological Technology Co., Ltd., China, AR1022) was used to show color, and positive results ranged from yellow to brownish yellow. The nucleus of hematoxylin staining (Beijing Solarbio Science & Technology Co., Ltd., China, Ltd, G1080) was blue. The tissue was then dehydrated, and microscopic examination, image acquisition, and analysis were performed. Three fields of view were randomly selected for each slice for statistical analysis.

### TUNEL staining

Paraffin sections were dewaxed in water and digested with protease K, and 3% hydrogen peroxide was added to block endogenous peroxidase. Add Labeling Buffer, 5% BSA, the Anti-DIG-Biotin, and SABC (Boster Biological Technology Co., Ltd., China, MK1015) in sequence. DAB (Boster Biological Technology Co., Ltd., China, AR1022) was used for color development, and the color of the DAB positive reaction was brown-yellow; the nucleus appeared blue after hematoxylin staining. The tissue was then dehydrated, and microscopic examination, image acquisition, and analysis were performed. Three fields of view were randomly selected for each slice for statistical analysis.

### Statistical analysis

The statistical analysis software was Stata/SE 16.0 All data are expressed as means ± standard deviation ($\overline{X }$ ± s). One-way ANOVA (LSD) was used to analyze the baseline data of each eye of the two groups of kittens, including axial length, diopter, and P100 wave of each eye. One-way ANOVA (LSD) was used to compare P100 waves in the same group at different times and in different groups at the same time. Paired sample *t*-test was used to compare the differences between different eyes in the same group, two independent sample *t*-test was used to compare the difference between the right eye in the control group and the experimental group. The results of IHC, ISH, and TUNEL were tested by independent sample *t*-test. Pearson correlation coefficient analysis was performed on the IHC, ISH, and TUNEL data of the control group and the experimental group.

## Results

### Baseline condition

There was no significant difference in diopter (*F* = 0.483, *P* = 0.696), axial length (*F* = 0.509, *P* = 0.679), amplitude (*F* = 0.013, *P* = 0.998) and latency (*F* = 0.629, *P* = 0.601) of PVEP between the two groups at 3 weeks of age (Table 1).

**Table 1:** Comparison of baseline situation between two groups of three-week-old kittens. ($\overline{X }$ ± *S*)

Group	Right eye of the experimental group	Left eye of the experimental group	Right eye of the control group	Left eye of the control group	*F*	*P*
Axial length (mm)	12.08 ± 0.32	12.07 ± 0.23	12.05 ± 0.202	12.19 ± 0.32	0.509	0.679
Diopter (D)	2.93 ± 0.49	3.00 ± 0.41	2.83 ± 0.50	2.78 ± 0.43	0.483	0.696
P100 latency (ms)	118.66 ± 3.10	118.36 ± 4.11	117.04 ± 2.41	117.27 ± 2.85	0.629	0.601
P100 amplitude (μV)	4.995 ± 0.661	5.073 ± 0.854	5.027 ± 1.033	5.051 ± 1.081	0.013	0.998

### P100 wave of PVEP

At the age of 3 weeks, there was no statistical difference in latency (*F* = 0.629, *P* = 0.601) and amplitude (*F* = 0.013, *P* = 0.998) of P100 wave between the right eye of the experimental group, the left eye of the experimental group, the right eye of the control group and the left eye of the control group. With the increase of age, the latency of four groups of P100 waves showed a downward trend, while the amplitude showed an upward trend. Moreover, although the latency and amplitude of the P100 wave in the right eye of the experimental group also showed a changing trend, the overall change was lower than that of the other three groups.

At postnatal age 5, 6, and 7 weeks, statistical differences were observed in latency and amplitude of P100 wave among the four groups. The latency of the P100 wave in the right (covered) eye of the experimental group was significantly higher than that in the left eye of the experimental group, the right eye, or the left eye of the control group (for *F* and *P* values, please see Tables 2 and 3). However, the amplitude of the P100 wave in the right (covered) eye of the experimental group was significantly lower than that in the left eye of the experimental group, the right eye, or the left eye of the control group (for *F* and *P* values, please see Tables 2 and 3).

**Table 2:** P100 latency in each group. ($\overline{X }$ ± *S*, ms)

Time (weeks)	Right eye of the experimental group	Left eye of the experimental group	Right eye of the control group	Left eye of the control group	*F*	*P*
3	118.66 ± 3.10	118.36 ± 4.11	117.04 ± 2.41	117.27 ± 2.85	0.629	0.601
4	113.00 ± 3.01	110.22 ± 3.52	108.84 ± 3.45*	109.50 ± 3.34*	3.005	0.043
5	109.22 ± 2.89	105.12 ± 2.04*	104.29 ± 2.52*	103.07 ± 4.48*	7.306	0.001
6	106.55 ± 3.90	101.03 ± 2.86*	99.11 ± 3.76*	99.08 ± 4.51*	8.593	< 0.001
7	104.80 ± 3.94	98.45 ± 3.82*	96.69 ± 4.20*	97.59 ± 3.83*	8.691	< 0.001
*F*	26.546	55.777	59.068	44.393		
*P*	< 0.001	< 0.001	< 0.001	< 0.001		

^*^Compared with the right eye of the deprivation group, there was a difference (*P* < 0.05)

**Table 3:** P100 amplitude in each group. ($\overline{X }$ ± *S*, μV)

Time (weeks)	Right eye of the experimental group	Left eye of the experimental group	Right eye of the control group	Left eye of the control group	*F*	*P*
3	4.995 ± 0.661	5.073 ± 0.854	5.027 ± 1.033	5.051 ± 1.081	0.013	0.998
4	6.413 ± 0.819	6.909 ± 1.066	7.274 ± 0.782*	7.274 ± 0.832*	2.139	0.112
5	7.138 ± 0.681	7.936 ± 0.805*	8.139 ± 0.820*	8.097 ± 0.775*	3.674	0.021
6	7.464 ± 0.907	8.730 ± 0.652*	8.897 ± 1.180*	8.880 ± 1.273*	4.467	0.009
7	7.687 ± 0.927	9.109 ± 0.908*	9.181 ± 0.996*	9.244 ± 0.941*	6.275	0.002
*F*	18.178	34.946	29.418	28.011		
*P*	< 0.001	< 0.001	< 0.001	< 0.001		

^*^Compared with the right eye of the experimental group, there was a difference (*P* < 0.05)

**Fig. 2:**
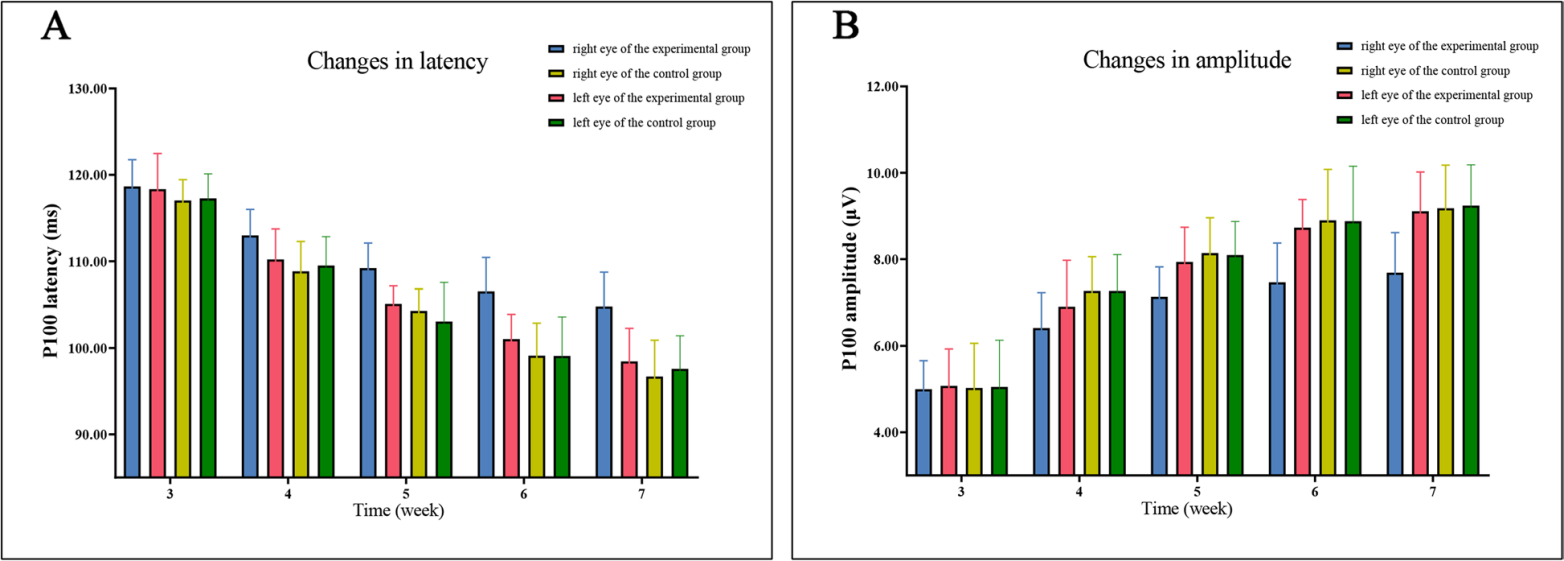
The latency and amplitude trend of P100 waves in 3-week-old to 7-week-old kittens. With the increase of age, the latency showed an upward trend (**A**) and the amplitude of each group showed a downward trend (**B**). At 5 and 7 weeks of age, the latency and amplitude of the right eye in the experimental group were statistically different from those in the other three groups

**Fig. 3:**
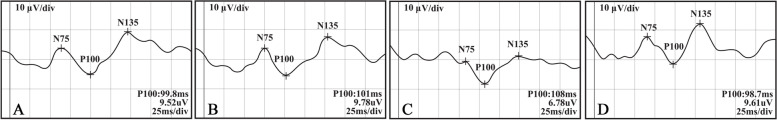
PVEP waves of each group of kittens after covering the right eye of the experiential group for 4 weeks. The latency in the right eye of the experimental group (**C**) was longer, and the amplitude was lower than in the right eye of the control group (**A**), the left eye of the control group (**B**), and the left eye of the experimental group (**D**)

The results showed that after 5 weeks of age, monocular form deprivation amblyopia had formed in the right eye of kittens in the experimental group (Figs. 2 and 3, Tables 2 and 3). (relevant data is available at https://figshare.com/s/41efd7f80329308c0d0a).

### Immunohistochemical staining

**Fig. 4:**
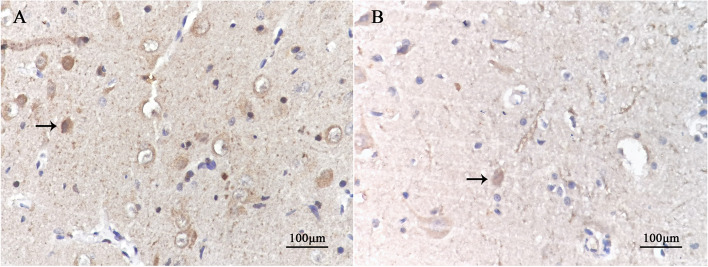
Immunohistochemical performance in the lateral geniculate body in each group (DAB X400). The black arrow in the picture indicates positive cells. The protein of ARC/Arg3.1 positive expression in the cytoplasm of neurons was brown-yellow. At the age of 7 weeks, there were more positive cells in the control group (**A**) and fewer in the experimental group (**B**)

The results of IHC showed that ARC/Arg3.1 protein was expressed in sections of the experimental group and the control group. ARC/Arg 3.1 protein expression in the cytoplasm, which was brown-yellow, and the nucleus was blue. At the age of 7 weeks, the average optical density of positive cells in the experimental group was lower than that in the control group (*P* < 0.001). The number of positive cells in the experimental group was lower than that in the control group (*P* < 0.001) (Fig. 4, Table 4) (relevant data is available at https://figshare.com/s/41efd7f80329308c0d0a).

**Table 4:** ARC/Arg3.1 immunohistochemical results of visual cortex in each group. ($\overline{X }$ ± *S*)

Group	Mean optical density of positive	Positive cell number
Experimental group	0.009294 ± 0.002941	19.10 ± 7.60
Control group	0.017041 ± 0.004765	34.83 ± 11.64
*t*	-7.579	-6.199
*P*	< 0.001	< 0.001

### In situ hybridization staining

**Fig. 5:**
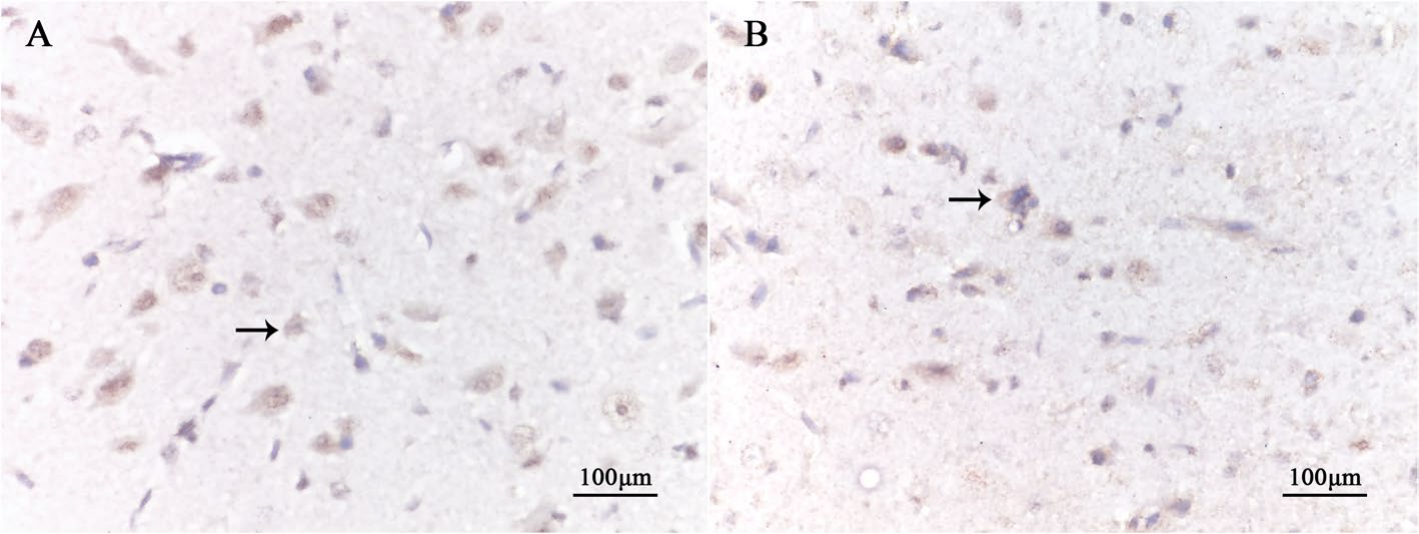
In situ hybridization performance in the lateral geniculate body in each group (DAB X400). The black arrow in the picture indicates positive cells. The protein of ARC/Arg3.1 positive expression in the cytoplasm of neurons was brown-yellow. At the age of 7 weeks, there were more positive cells in the control group (**A**) and fewer in the experimental group (**B**)

The results of ISH showed that ARC/Arg3.1 mRNA was expressed in sections of the experimental group and the control group. ARC/Arg 3.1 mRNA expression in the cytoplasm, which was brown-yellow, and the nucleus was blue. At the age of 7 weeks, the average optical density of positive cells in the experimental group was lower than that in the control group (*P* < 0.001). The number of positive cells in the experimental group was lower than that in the control group (*P* < 0.001) (Fig. 5, Table 5) (relevant data is available at https://figshare.com/s/41efd7f80329308c0d0a).

**Table 5:** ARC/Arg3.1 in situ hybridization results of visual cortex in each group. ($\overline{X }$ ± *S*)

Group	Mean optical density of positive	Positive cell number
Experimental group	0.006526 ± 0.001905	43.07 ± 11.52
Control group	0.013550 ± 0.004042	58.33 ± 14.53
*t*	-8.609	-4.508
*P*	< 0.001	< 0.001

### TUNEL staining

**Fig. 6:**
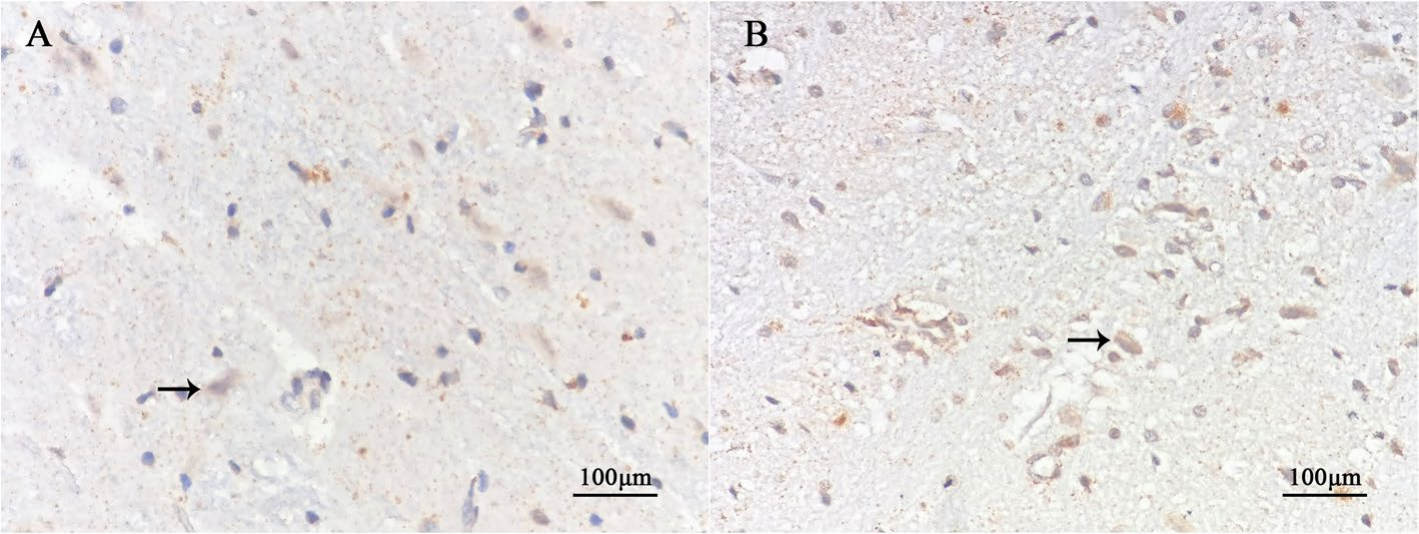
TUNEL performance in the lateral geniculate body in each group (DAB X400). TUNEL-positive cells were brown-yellow, while TUNEL-negative cells were blue. The black arrow in the picture indicates the positive cells. At 7 weeks of age, there were fewer TUNEL-positive cells in the control group (**A**) and more TUNEL-positive cells in the experimental group (**B**)

The results of TUNEL showed that there were positive cells in the slices of the experimental group and the control group. The nucleus of positive cells was brown-yellow, while that of negative cells was blue. At the age of 7 weeks, the average optical density of positive cells in the experimental group was higher than that in the control group (*P* < 0.001). The number of positive cells in the experimental group was more than that in the control group (*P* < 0.001) (Fig. 6, Table 6) (relevant data is available at https://figshare.com/s/41efd7f80329308c0d0a).

**Table 6:** Tunel staining results in each group. ($\overline{X }$ ± *S*)

Group	Mean optical density of positive	Positive cell number
Experimental group	0.005364 ± 0.001867	45.73 ± 16.48
Control group	0.003595 ± 0.001586	27.07 ± 14.09
*t*	3.954	4.715
*P*	< 0.001	< 0.001

### The analysis of the correlation

Pearson correlation coefficient indicated that the intensity of TUNEL positive reaction was negatively correlated with the mean optical density of positive cells from ARC/Arg3.1 protein (*PCCs* = -0.415, *P* = 0.001) and mRNA (*PCCs* = -0.409, *P* = 0.001) expression at the age of 7 weeks, and ARC/Arg3.1 protein expression was positively correlated with ARC/Arg3.1 mRNA expression (*PCCs* = 0.958, *P* < 0.001) (Table 7).

**Table 7:** The analysis of correlation analysis among ISH, IHC and TUNEL. (*PCCs*, *P*)

Group	IHC	ISH	TUNEL
	Mean optical density of positive	Mean optical density of positive	Mean optical density of positive
ISH	0.958, < 0.001	-	-0.409, 0.001
Tunel	-0.415, 0.001	-0.409, 0.001	-

## Discussion

The application of kittens as models of formal deprivation amblyopia can be traced back to the studies of Wiesel & Hubel (1963) [16] and Hubel & Wiesel (1970) [17]. Some studies have shown that for kittens, the effect of form deprivation on vision usually peaks from birth to 4 weeks old. After 12 weeks of age, the form deprivation caused by occlusion can hardly interfere with their visual development [12, 17, 18]. Therefore, in this study, 3-week-old kittens were selected, which can fully ensure that their covering eyes are affected by form deprivation factors during the visual development period of kittens, and have maximum interference on their visual development, to successfully establish a monocular form deprivation amblyopia kitten model. Some studies have shown that when the eyes of kittens are covered by monocular deprivation, the transformation of the ocular dominance column can occur in a short time [19, 20]. With the prolongation of deprivation time, the deviation of dominant column pairs will become more obvious.

To observe the form deprivation eyes of kittens and compare whether form deprivation amblyopia is formed. In this study, the results of PVEP detection were used as the basis for the establishment of the amblyopia model. At present, it has been mature and applied to the establishment of the amblyopia animal model [21]. The retina is stimulated by external light, which will produce potential changes and form nerve impulses. Nerve impulses pass through the optic nerve, optic chiasma, optic bundle, lateral geniculate body, and optic radiation in turn, and finally pass to the visual center of the cerebral cortex. Under the condition of normal visual development of kittens, the conduction velocity and mode of cells related to vision can stimulate consistent potential activity, form harmonious synchronous oscillation in front of and visual cortex, and then produce a regular waveform [22].

The results showed that with the development of the visual system of kittens, after 4 weeks of covering, the latency of the P100 wave decreased and the amplitude increased in both eyes of the experimental group and control group. However, in the experimental group of the right eye compared with the other three groups, although there is a certain degree of change, the overall range is still lower than the other three groups. Moreover, after 2 weeks of occlusion (at the fifth week of age), the latency of the P100 wave in the occluded eyes of the experimental group was significantly higher than that of the other three groups, and the amplitude of the P100 wave was significantly lower than that of the other three groups. Therefore, we believe that form deprivation amblyopia developed in the right eye of the experimental group at the fifth week of age. This result is consistent with the results of the previous [23, 24].

The balance of excitation and depression at the axonal level of the visual cortex is the condition of maintaining the normal development and function of the visual cortex, and it is also an important factor affecting the plasticity of the visual system [25]. The first synaptic replacement of optic nerve impulse in the brain occurs in the lateral geniculate body, and each lateral geniculate body receives projections from temporal optic nerve fibers of the ipsilateral retina and nasal optic nerve fibers of the contralateral retina, and this projection has strict local regional correspondence. As a semi-crossover animal, the optic nerve fibers of kittens pass through the optic chiasma to the lateral geniculate body, and the lateral geniculate body of kittens can be divided into A, A1, C, and C1-C3 layers. Layers A, C, and C2 received projections from contralateral ocular fibers, while layers A1 and C1 received projections from ipsilateral ocular fibers. Some studies have found that C-fos, GABA, and brain-derived neurotrophic factor (BDNF) in the contralateral lateral geniculate body of the amblyopic eyes are down-regulated compared with those in the ipsilateral amblyopic eyes in kittens with monocular form deprivation amblyopic eyes [26–28].

Synapse, as the structural basis of information transmission between neurons, is the key part of visual development plasticity. Some studies have suggested that synaptic plasticity is considered the most critical link in the pathogenesis of amblyopia [29]. Neural storage in neural networks is considered to depend partly on the plasticity of synapses [30, 31]. Synaptic plasticity refers to the change of synaptic connections between neurons when synapses are in use or disuse [31, 32]. In terms of time, it can be divided into two types: LTP and LTD [33]. LTP is a kind of information storage mode at the synaptic level, and the induction mechanism of LTP is mainly the Ca2^+^ influx caused by the change of the NMDAR channel in the postsynaptic membrane, which triggers a series of biochemical processes in cells. LTD refers to activity-dependent persistent potential attenuation induced on an unstimulated pathway, which is the opposite of LTP. For example, the visual cortex response caused by visual deprivation is an LTD phenomenon.

Considering the induction and expression mechanism of LTP and LTD in the lateral geniculate body, it is similar to the hippocampus [34–38]. For example, NMDAR needs to be activated, which leads to the activation of a series of intracellular kinases and the redistribution of AMPA receptors. The first change in the plasticity of the lateral geniculate body is synaptic efficiency, which does not need to synthesize new proteins, and then there will be long-term changes in neural pathways. However, long-term changes in neural pathways require gene expression and new protein synthesis, so kinase activation will lead to gene expression, which may be realized through the activation of transcription factors. Some studies have shown that NMDAR is related to the changes in the visual cortex in amblyopia animals [39, 40]. However, Ziburkus et al. [41] found that the expression of NMDAR in the lateral geniculate body of amblyopia animals did not seem to be affected. This is contrary to some research results in recent years.

Arc/arg3.1 encodes a protein of about 400 amino acids, which has no catalytic or other known functional motifs [42–44]. Arc/Arg3.1 protein interacts directly or indirectly with many proteins, indicating that it has the function of a pivotal protein [45–49]. Most of its functions are thought to occur at postsynaptic sites. Biochemical and electron microscopic studies show that Arc/Arg3.1 protein exists in the postsynaptic density of activated neurons [50–52]. As an archetypal immediate-early gene, ARC/Arg3.1 is generally considered a reliable marker of neuronal activity [53, 54]. ARC/Arg3.1 is also necessary for various forms of learning and memory, and some studies suggest that it is related to synaptic plasticity [44]. For example, ARC/Arg3.1 could regulate the expression of AMPAR during homeostatic plasticity with LTD and then maintain LTP [55–57]. ARC/Arg3.1 plays a key role in the long-term synaptic plasticity of excitatory synapses and memory and postnatal cortical development [58, 59]. These results suggest a physiological function of Arc in enhancing the overall orientation specificity of visual cortical neurons during the post-eye-opening life of an animal. Wang et al. [60] showed a physiological function of Arc in enhancing the overall orientation specificity of visual cortical neurons.

Some studies have shown that LTP and LTD, heterosynaptic LTD (inverse synaptic tagging), and homeostatic scaling all require the synthesis of ARC/Arc3.1, but the specific mechanism that determines the prominent changes is still unclear [58]. When BDNF was injected into the hippocampus, researchers found that a transcription-dependent LTP induced by ARC/Arg3.1 mRNA in the granulosa cell body and dendrite can be induced [61, 62]. Another study shows that the expression of BDNF is associated with the maintenance of high-frequency stimulation long-term potential (HFS-LTP) [57]. Injection of Arc antisense oligodeoxynucleotides (Arc-AS) before injection of BDNF inhibits the induction of LTP, which indicates that the process is completely downstream of the BDNF signaling pathway [57, 63]. After injecting BDNF for 2 h, the LTP can be restored to the baseline level by injecting Arc-AS (Arc antisense oligodeoxynucleotides) again. However, after injecting BDNF for 4 h, Arc-AS injection has no effect [57]. Therefore, some researchers believe that the newly synthesized ARC/Arg 3.1 can regulate the expression and consolidation of LTP induced by HFS-LTP and exogenous BDNF [58] In addition, Qi et al. [64] showed that ARC/Arg3.1 can be activated and up-regulated by PKA/CREB and ERK/CREB signaling pathways and they found a significant increase in the number of neuronal apoptosis in the model group after ARC/Arg 3.1 gene knockout.

Based on these studies and our results, we speculate that the number of neuronal apoptosis in the lateral geniculate body increases due to the influence of form deprivation. The change of neuron number in the lateral geniculate body further leads to the decrease of ARC/Arg3.1 expression, which leads to abnormal activities such as LTP and LTD, and finally promotes the further development of amblyopia.

However, this study still has some limitations. We proved that the expression protein and mRNA of ARC/Arg3.1 were down-regulated in the visual cortex of amblyopia kittens, but we failed to detect the dynamic change of ARC/Arg3.1 or set different groups to observe its changes with age. On the other hand, we only compared the expression of ARC/Arg3.1 in the lateral geniculate body of amblyopia kittens and normal kittens and did not study the change of its expression in different layers of the lateral geniculate body. In addition, the statistics of slices in this study were carried out at high power using three random visual fields. Although we have adopted some methods to reduce the risk of bias as much as possible, this method still has some limitations.

## Conclusions

To sum up, the expression of ARC/Arg3.1 is associated with monocular form deprivation amblyopia and apoptosis of lateral geniculate body cells. This study speculates that ARC/Arg3.1 gene plays an important role in visual development.

## Data Availability

The datasets used and analyzed during the current study are available from the corresponding author upon reasonable request. Or all relevant datasets related to the study can be found in the specified (database.https://figshare.com/s/41efd7f80329308c0d0a).

## Abbreviations

ARC/Arg3.1: Activity-regulated cytoskeleton-associated
LTP: Long-term potentiation
LTD: Long-term depression
PVEP: Pattern visual evoked potential
IHC: Immunohistochemistry
ISH: In situ hybridization
TUNEL: TdT-mediated dUTP nick-end labeling
HFS-LTP: High-frequency stimulation long-term potential
Arc-AS: Arc antisense oligodeoxynucleotides
DAB: Diaminobenzidine
SABC: Strept avidin–biotin complex
